# In Silico and In Vitro Identification of P-Glycoprotein Inhibitors from a Library of 375 Phytochemicals

**DOI:** 10.3390/ijms241210240

**Published:** 2023-06-16

**Authors:** Julia Schäfer, Vincent Julius Klösgen, Ejlal A. Omer, Onat Kadioglu, Armelle T. Mbaveng, Victor Kuete, Andreas Hildebrandt, Thomas Efferth

**Affiliations:** 1 Department of Pharmaceutical Biology, Institute of Pharmaceutical and Biomedical Sciences, Johannes Gutenberg University, Staudinger Weg 5, 55128 Mainz, Germany; 2 Institute of Bioinformatics, Johannes Gutenberg University, 55131 Mainz, Germany; 3 Department of Biochemistry, Faculty of Science, University of Dschang, Dschang P.O. Box 67, Cameroon

**Keywords:** cancer, chemotherapy, molecular docking, multidrug resistance, natural products, virtual drug screening

## Abstract

Cancer therapy with clinically established anticancer drugs is frequently hampered by the development of drug resistance of tumors and severe side effects in normal organs and tissues. The demand for powerful, but less toxic, drugs is high. Phytochemicals represent an important reservoir for drug development and frequently exert less toxicity than synthetic drugs. Bioinformatics can accelerate and simplify the highly complex, time-consuming, and expensive drug development process. Here, we analyzed 375 phytochemicals using virtual screenings, molecular docking, and in silico toxicity predictions. Based on these in silico studies, six candidate compounds were further investigated in vitro. Resazurin assays were performed to determine the growth-inhibitory effects towards wild-type CCRF-CEM leukemia cells and their multidrug-resistant, P-glycoprotein (P-gp)-overexpressing subline, CEM/ADR5000. Flow cytometry was used to measure the potential to measure P-gp-mediated doxorubicin transport. Bidwillon A, neobavaisoflavone, coptisine, and z-guggulsterone all showed growth-inhibitory effects and moderate P-gp inhibition, whereas miltirone and chamazulene strongly inhibited tumor cell growth and strongly increased intracellular doxorubicin uptake. Bidwillon A and miltirone were selected for molecular docking to wildtype and mutated P-gp forms in closed and open conformations. The P-gp homology models harbored clinically relevant mutations, i.e., six single missense mutations (F336Y, A718C, Q725A, F728A, M949C, Y953C), three double mutations (Y310A-F728A; F343C-V982C; Y953A-F978A), or one quadruple mutation (Y307C-F728A-Y953A-F978A). The mutants did not show major differences in binding energies compared to wildtypes. Closed P-gp forms generally showed higher binding affinities than open ones. Closed conformations might stabilize the binding, thereby leading to higher binding affinities, while open conformations may favor the release of compounds into the extracellular space. In conclusion, this study described the capability of selected phytochemicals to overcome multidrug resistance.

## 1. Introduction

Cancer is the most common cause of death after cardiovascular diseases [1,2]. Older people are generally more susceptible to cancer, and cancer remains an important health issue worldwide as the aging of the population further increases. Although unprecedent efforts led to remarkable successes in cancer diagnostics and treatment, anticancer drugs are still prone to the development of resistance and significant toxicities, such as immunosuppression, specific organ damage, nausea, vomiting, and fever. Drug resistance represents a major challenge, as it limits the effectiveness of tumor therapy and frequently leads to treatment failures with fatal outcomes for the patients [3,4,5,6]. Hence, there is an urgent requirement for novel therapeutic agents with less resistance potential and higher specificity.

There are numerous mechanisms of drug resistance, i.e., alterations in tumor cells at the drug target sites, as well as mechanisms upstream and downstream thereof. Drug target site mechaisms include point mutations in target proteins, as well as DNA damage and problems with repair mechanisms, etc. Alterations upstream of the target sites are increased drug efflux or decreased drug influx at the cell membrane, increased metabolization and detoxification, i.e., by cytochrome P450 monooxygenases, etc. Finally, alterations downstream of the target sites are the inability to induce the programmed cell death, despite lethal drug action, e.g., defects in the apoptotic cascade [7,8].

One phenomenon has attracted attention for many years, which is characterized by a broad spectrum of cross-resistance to numerous clinically established drugs from diverse classes and with distinct functions, including classical anticancer drugs (e.g., anthracyclines, *Vinca* alkaloids, taxanes, antitumor antibiotics, epipodophyllotoxins), but also modern targeted chemotherapeutics (e.g., small molecular tyrosine kinase inhibitors). For this reason, this broad cross-resistance profile has been termed multidrug resistance (MDR). A major mechanism of MDR is a drug transporter belonging to the ATP-binding cassette (ABC) family, P-glycoprotein (P-gp). This membrane protein actively expels a large variety of harmful xenobiotic compounds that have passively diffused into cells [9,10]. The pumping activity of P-gp keeps the concentration of anticancer drugs at sublethal intracellular concentrations, which finally favors the survival of MDR cells and the clinical failure of chemotherapy.

There have been huge efforts to develop chemical inhibitors of P-gp’s efflux function [11]. It was seen, however, with great disappointment in the scientific community, that these synthetic compounds failed in clinical trials because of their undesired side effects [12,13].

For millennia, phytotherapy has been used in traditional medicines all over the world, and, still today, a majority of the global population, especially in low-and middle-income countries, relies on herbal recipes for primary health care [14]. It has been repeatedly reported that natural products, their derivatives, and their principles of bioactivity are relevant for drug treatment in Western academic medicine [15,16]. Since herbal medicines are considered to exert only moderate or few side effects, they are attractive not only for therapy, but also for the prevention of diseases [17,18]. Natural products have, therefore, also been discussed as a resource to develop drugs to combat resistance to established anticancer drugs [19,20].

In rational drug design, the identification of appropriate disease targets is crucial. Chemical lead structures binding to disease-relevant target proteins guide the preclinical drug development process. With regards to this concept, cheminformatics and bioinformatics provide powerful tools to identify novel drug candidates. An important part of rational drug discovery is computer-aided drug discovery, including virtual screenings, molecular docking, and in silico prediction of quantitative structure activity relationship (QSAR) and absorption, distribution, metabolism, excretion, and toxicity (ADMET) [21,22,23]. The aim of computer-based drug design is to find the best possible balance of all relevant pharmacological properties for a compound. Thus, an optimal drug would be very effective and highly selective for its target site, thereby exerting no or little effects on healthy organs and tissues in the body, which may lead to undesired side effects. In the past decades, the use of bioinformatic tools and programs has become a huge part of drug discovery, making the development progress more effective in terms of speed and costs [24,25].

In the present study, we performed in silico screenings of 375 natural compounds that have been investigated in our department during the past two decades. Based on these in silico results of the present analysis, we selected six candidate compounds for in vitro tests in terms of their inhibition of P-gp function. We recently reported a series of point mutations in the *ABCB1/MDR1* gene encoding P-gp of clinical tumor biopsies [26]. Therefore, we addressed the question of whether phytochemicals interact not only with wildtype P-gp, but also with a panel of 10 P-gp mutants with specific clinically relevant point mutations.

## 2. Results

**Figure 1:**
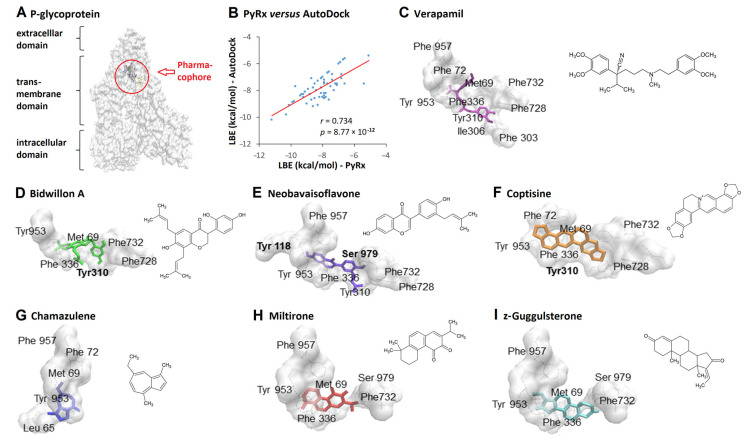
Virtual screening and molecular docking of 375 phytochemicals to P-glycoprotein. (**A**) Three-dimensional model of P-glycoprotein with the drug-binding site (pharmacophore) at the inner channel of the transmembrane domain. (**B**) Linear regression and Pearson correlation of the top 20 compounds with the lowest binding energies (LBE) obtained from PyRx and AutoDock 4.2 from each of the classes of flavonoids, alkaloids, and terpenes. (**C**) Binding position of verapamil, a well-known P-glycoprotein inhibitor with amino acid residues involved in drug-binding. (**D**–**I**) Binding positions of six selected candidate phytochemicals with amino acid residues involved in drug-binding.

We first screened 375 phytochemicals for their lowest binding energies (LBE) to P-glycoprotein (P-gp). This virtual screening was performed with PyRx software in a blind docking mode. Then, we validated these results by performing molecular docking using AutoDock 4.2 program in a defined mode choosing the drug-binding site at the inner channel of P-gp’s transmembrane region (Figure 1A). We obtained LBE values in a range from −10.8 to −7.2 kcal/mol for flavonoids, from −11.4 to −5.1 kcal for alkaloids, and from −11.2 to 5.0 kcal/mol for terpenes. The PyRx- and AutoDock-derived LBE values for the top 20 compounds from each of the classes of flavonoids, alkaloids, and terpenes were subjected to linear regression and Pearson correlation analyses. The LBE values obtained by both methods significantly correlated (*r* = 0.734; *p* = 8.77 × 10^−12^) (Figure 1B).

Molecular docking provides information on the interaction of a compound with a target protein but cannot distinguish whether a compound serves as substrate or acts as inhibitors for P-gp. Therefore, Torch3D analysis was used for structural and electrostatic alignment of the 375 phytochemicals with a panel of known Pgp-inhibitors, i.e., clomipramine, verapamil, lidocaine, propanalol, tamoxifen, and trifluoperazine. A high score represents a high accordance of the electrostatic fields around the molecules. Compounds with high scores for several reference molecules were determined.

The Derek nexus module from the StarDrop module was used to predict the toxic potential of the compounds. Among the alkaloids, 31 compounds showed severe toxicities. The most common toxicities were HERG channel inhibition (indicating cardiotoxicity) and chromosome damage (indicating genotoxicity). A total of 72 flavonoids showed possible toxicities. Most predicted toxicities of flavonoids comprised were chromosome damage, mutagenicity, and estrogen receptor modulation. Terpenes mostly showed chromosome damage, mutagenicity, and hepatotoxicity for a total of 72 compounds. Furthermore, 61 alkaloids, 35 flavonoids, and 103 terpenes revealed no toxic potential.

**Figure 2:**
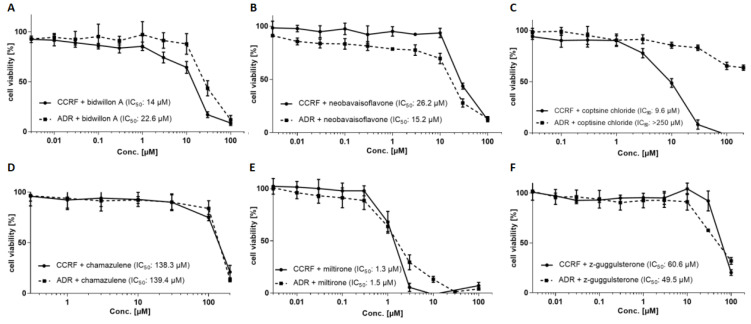
Dose–response curve for the nine selected compounds. (**A**) bidwillon A; (**B**) neobavaisoflavone; (**C**) coptisine chloride; (**D**) chamazulene; (**E**) miltirone; (**F**) z-guggulsterone. Shown are the dose–response curves of wildtype drug-sensitive CCRF-CEM cells and their P-gp-overexpressing multidrug-resistant subline CEM/ADR5000. Mean values ± SD of three independent experiments, each with six parallel measurements, are presented.

Based on all in silico results above, six promising compounds were chosen according to the following criteria: low LBE values, no predicted toxicity, and multiple Torch3D high scores (>0.5) in alignment with known P-gp inhibitors. Among the flavonoids, bidwillon A and neobavaisoflavone were chosen. Cationic coptisine was selected from the class of alkaloids, and miltirone, chamazulene, and z-guggulsterone were chosen from the group of terpenes. The molecular dockings and chemicals strcutures of thse compounds are shown in Figure 2D–I. Verapamil served as positive control for a P-gp inhibitor (Figure 1C). Table 1 summarizes the calculated LBE values and interacting amino acid residues of the six chosen compounds, as well as the highest scores in Torch3D screening.

The growth-inhibitory activities of the six candidate compounds were detected by the resazurin assay (Figure 2). Bidwillon A inhibited CCRF-CEM and CEM/ADR-5000 cells with IC_50_ values of 14.0 ± 1.6 µM and 22.6 ± 1.3 µM, respectively, indicating a weak cross-resistance (1.8-fold) (Figure 2A). Neobavaisoflavone showed collateral sensitivity in multidrug-resistant cells. The IC_50_ values of neobavaisoflavone were 26.2 ± 1.7 µM and 15.2 ± 2.4 µM for CCRF-CEM and CEM/ADR-5000 cells, respectively (Figure 2B). The multidrug-resistant CEM/ADR5000 cells were cross-resistant to coptisine chloride. The IC_50_ values were 9.6 ± 1.3 µM for CCRF-CEM and >250 µM for CEM/ADR5000 cells (Figure 2C). Miltirone inhibited CCRF-CEM cells (IC_50_: 1.3 ± 0.3 µM) and CEM/ADR5000 cells (IC_50_: 1.5 ± 0.03 µM) (Figure 2D). Chamazulene inhibited both cell lines with comparable activities. The IC_50_ values were 138.3 ± 7 µM and 139.4 ± 6.9 µM for sensitive and multidrug-resistant cells (Figure 2E). The multidrug-resistant cells were collaterally sensitive to Z-guggulsterone. The IC_50_ values ranged from 60.6 ± 6.1 µM for CCRF-CEM cells to 49.5 ± 3.7 µM for CEM/ADR5000 cells (Figure 2F).

**Figure 3:**
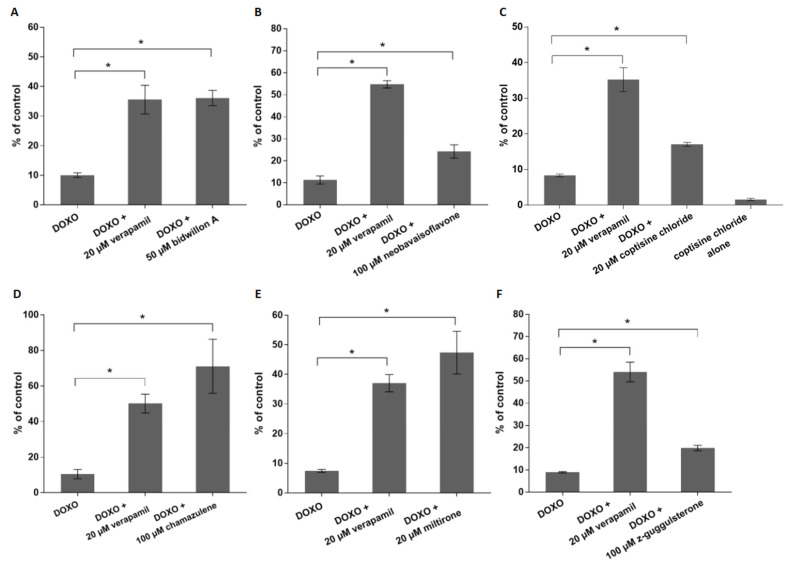
Flow cytometric analysis of doxorubicin uptake by P-gp-overexpressing CEM/ADR5000 cells with and without coincubation of six phytochemical candidate compounds. The bar diagrams represent mean fluorescence intensity values of CEM/ADR5000 cells as percentages of the fluorescence in CCRF-CEM control cells. The bars show mean values ± SD of three independent experiments. (**A**) bidwillon A; (**B**) neobavaisoflavone; (**C**) coptisine chloride; (**D**) chamazulene; (**E**) miltirone; and (**F**) z-guggulsterone. * *p* < 0.05.

Figure 3 depicts the doxorubicin uptake in CEM/ADR5000 cells treated with the selected six phytochemicals in comparison to treatment with verapamil as a known P-gp inhibitor. Upon treatment with bidwillon A, the doxorubicin uptake significantly increased compared to cells treated with doxorubicin alone (*p* = 0.05). The effect of bidwillon A was comparable to that of verapamil (Figure 3A). Although the fluorescence intensity significantly increased in cells treated with neobavaisoflavone or z-guggulsterone plus doxorubicin compared to cells treated with doxorubicin only, this effect was weaker than that of verapamil (Figure 3B,F). Figure 3D,E show the increased doxorubicin fluorescence in CEM/ADR5000 cells treated with doxorubicin in combination with chamazulene or miltirone. Remarkably, this effect was even stronger than that of verapamil.

Supplementary Figure S1 exemplarily depicts histograms of doxorubicin uptake experiments using flow cytometry. Here, we used coptisine chloride as the candidate compound to inhibit P-gp and, thereby, we increased intracellular doxorubicin accumulation. The autofluorescence of CEM/ADR5000 cells is very low and visible at the very left of Supplementary Figure S1 (marked in red). The fluorescence of CEM/ADR5000 cells treated with doxorubicin was higher (blue color), but it was much less than the fluorescence of CCRF-CEM cells incubated with doxorubicin (yellow color), indicating that CEM/ADR5000 accumulated much less doxorubicin than CCRF-CEM cells due to P-gp-mediated doxorubicin transport out of the cells. Inhibition of P-gp by verapamil (control drug) or coptisine chloride yielded a considerable fluorescence shift to the left (green and pink colors). It should be noted that coptisine chloride alone also produced a fluorescence signal (orange color). Hence, this fluorescence signal may influence the measurement of doxorubicin fluorescence. Coptisine chloride was the only of the six candidate compounds that exerted autofluorescence.

**Figure 4:**
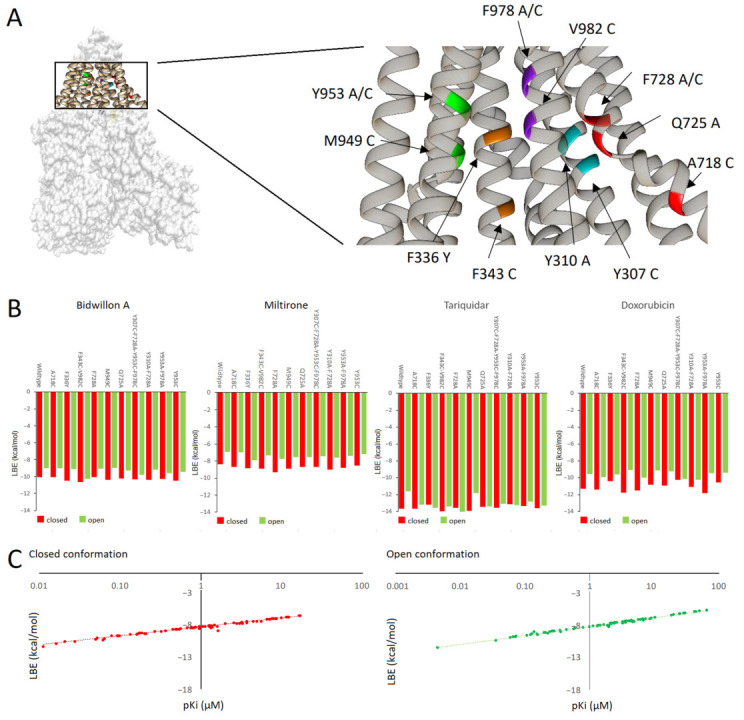
Molecular docking of bidwillon A and miltirone in relation to wildtype and mutated P-gp forms. (**A**) Missense mutations in the transmembrane domains of P-gp. (**B**) Molecular docking (defined mode) of bidwillon A and miltirone in relation to single, double, and quadruple P-gp mutants. Tariquidar and doxorubicin served as control drugs as the known inhibitor and the substrate, respectively. (**C**) Linear regressions of all binding energies and predicted inhibition constant (pKi) values in closed and open P-gp conformations (*r* > 0.9).

For further analyses regarding P-gp mutants, we selected bidwillon A and miltirone. We generated P-gp homology models in closed and open confirmations, each harboring six single missense mutations (F336Y, A718C, Q725A, F728A, M949C, Y953C), three double mutations (Y310A-F728A; F343C-V982C; Y953A-F978A), or one quadruple mutation (Y307C-F728A-Y953A-F978A) in the transmembrane region (Figure 4A).

These P-gp models have been used for defined molecular docking at the drug-binding site. The binding energies for bidwillon A and miltirone, together with tariquidar and doxorubicin, as the known inhibitor and the substrate, respectively, are shown in Figure 4B. In general, major differences between the binding energies between the wildtype and mutated P-glycoproteins have not been observed. Another common feature was that the binding affinities of the compounds were slightly better or equal in the closed compared to the open conformations. The lowest binding energies (LBE) of bidwillon A were comparable to those of doxorubicin, while those of tariquidar were lower, and those of miltirone were higher (Figure 4B). Furthermore, there was a tight relationship between the LBE values and the predicted inhibition constants (pKi) for all compounds in both closed and open conformations of wildtype and mutated P-glycoproteins (Figure 3C).

## 3. Discussion

During the past three decades, there have been huge efforts to develop chemical P-gp inhibitors for clinical use. Unfortunately, these efforts have gone without success regarding the clinical implemenation of P-gp inhibitors into routine treatment protocols. Alternative concepts regarding the development of synthetic small molecules, such as P-gp efflux inhibitors, have been proposed, including the use of nanotechnological devices, compounds that exert collateral sensitivity of multidrug-resistant cells, and natural products [27,28,29,30,31]. Regarding natural products, there have been not only considerable efforts to identify P-gp efflux inhibitors in vitro, but also in vivo, indicating that natural products may indeed have the potential to overcome multidrug resistance in living organisms, although clinical trials are still missing [32,33,34].

In the present investigation, we investigated a chemical library of 375 phytochemicals representing a collection of compounds that have been used in our laboratory during the past two decades. We have chosen compounds that represent a broad chemical diversity to avoid a possible bias by restricting the chemical diversity. At the beginning of this investigation, it seemed less clear whether alkaloids are better inhibitors than terpenes or flavonoids, or vice versa. It is also known, from a huge number of investigations with synthetic P-gp inhibitors, that clear chemical principles for the chemical structures of efficient P-gp inhibitors (and substrates as well) could not be defined, since P-gp is a “promiscuous” transporter that expels a large variety of chemically diverse molecules. We anticipate that this is not only true for synthetic compounds, but it could also be true for natural products. One reason for this phenomenon may be the “induced fit” features of P-gp, i.e., the pharmacophore exhibits a large flexibility in binding and expelling compounds [35].

Analyzing these chemicals structures for virtual screening and molecular docking to P-gp, we focused on six candidate compounds for subsequent in vitro studies. Computer-based screening approaches represent powerful tools in early drug discovery. Bioinformatical tools act as trend-setters for the prediction of valuable chemical lead structures in a time- and cost-saving manner [36]. The six candidate compounds were two flavonoids (bidwillon A, neobavaisoflavone), one alkaloid (coptisine), and three terpenes (chamuzulene, miltirone, and z-guggulsterone). CEM/ADR5000 cells were strongly cross-resistant to coptisine chloride and weakly cross-resistant to bidwillon A, sensitive to chamazulene and miltirone, and collaterally sensitive to neobavaisoflavone and z-guggulsterone. The uptake of doxorubicin in CEM/ADR5000 cells was strongly enhanced by bidwillon A, chamazulene, and miltirone, and it was weakly enhanced by neobavaisoflavone, coptosine chloride, and z-guggulsterone. In a previous investigation, we described that P-gp(MDR1/ABCB1) was 448-fold up-regulated due to 7q21.12 amplification in CEM/ADR5000 compared to the parental CCRF-CEM cell line [37]. Although other resistance mechanisms may also be operative in CEM/ADR5000 cells, P-gp is by far the most predominant one. Therefore, we think that the influence of other mechanisms is minor compared to P-gp.

Flavonoids, alkaloids, and terpenes are commonly found in fruits, vegetables, and medicinal herbs as plant secondary metabolites. A variety of bioactivities have been reported for flavonoids, such as anti-bacterial, anti-allergic, and anti-oxidative properties, among others. Due to their potential pharmacological properties and rather low toxicities, they are widely used as drug and dietary supplements.

Bidwillon A is isolated from the bark of *Erythrina sigmoidea* and *Bituminaria morisiana* and has been reported for its antibacterial activity against *E. coli* and methicillin-resistant *Staphylococcus aureus* [38,39]. It also inhibited leukemia cells [40,41]. The in vivo activity of this compound has not been reported yet.

Neobavaisoflavone is isolated from *Psoralea corylifolia* and is known for its senescence-associated secretory phenotype (SASP) [42] and its anti-inflammatory and anti-osteoarthritic activities [43,44,45]. Additionally, it displayed antibacterial activity against Gram-negative multidrug-resistant phenotypes, expressing active efflux pumps [39]. This compound inhibited murine and human lung and glioma tumors in vivo [46,47,48]. It underwent intensive metabolism in vivo [49].

Coptisine is an alkaloid found in Chinese Goldthreat (*Coptis chinensis*). It was extracted from Coptidis Rhizoma and possesses anti-inflammatory, anti-bacterial, and anticancer activities [50]. This compound suppressed the expression of inducible NO synthase (iNOS) and cyclooxygenase-2 (COX-2), as well as the IL-1β-induced inflammatory response in osteoarthritis by suppressing the NF-κB signaling pathway [51]. The anticancer activity of coptisine has been shown in vitro and in vivo [52,53].

Chamazulene is a main aromatic compound extracted from chamomile (*Matricaria chamomilla*). It appears as a deep blue oil and is widely used in herbal remedies and cosmetic products [54]. It exerted anti-inflammatory properties in several animal models and inhibited CYP1A2. Furthermore, it inhibited the formation of leukotriene B4 in intact cells and is an inhibitor of cyclooxygenase-2 [55]. Thus far, the anticancer activity of chamazulene has been reported as part of essential oils or extracts of *Artemisia* species [56].

Miltirone was isolated from the roots of Chinese medical herb *Salvia miltiorrhiza.* The compound possesses numerous pharmacological activities, including prevention of angina pectoris, myocardial infarction, and cancer [57]. It induced apoptosis by triggering ROS-mediated MAP-kinase signaling pathways and activating caspases in wildtype and P-gp-overexpressing human HepG2 hepatoma cell lines and inhibited human leukemia xenograft growth in vivo [57,58]. Together with our results on multidrug-resistant CEM/ADR5000 leukemia cells, and, thus, accordingly, miltirone might be a promising compound to treat multidrug-resistant cancers.

Z-Guggulsterone is an active constituent of the Indian medical plant *Commiphora mukul* that has been used in Ayurveda for the treatment of many disorders [59]. This compound inhibited angiogenesis [60], increased the transcription of bile salt export pumps, and regulated cholesterol homeostasis [61]. Z-guggulsterone inhibited not only cancer growth in vivo [60,62], but it also reversed doxorubicin resistance [63]. Among these six candidate compounds, we favored bidwillon A and miltirone for further analyses. Our molecular docking indicated interactions between the selected phytochemicals and P-glycoprotein, but we cannot give functional clues. Therefore, additional in vitro techniques are necessary to verify and specify the nature of interactions between compounds and target proteins, i.e., whether the compounds are substrates or inhibitors of P-gp. For this reason, we applied the resazurin assay and a flowcytometric doxorubicin uptake assay. We estimated a compound as a P-gp substrate if multidrug-resistant cells are cross-resistant to this phytochemical in the resazurin assay. Since doxorubicin is not only a well established anticancer drug and P-gp substrate, but also exerts strong fluorescent properties, it can be used as an exquisite probe for uptake studies. MDR cells take up doxorubicin much less than sensitive cells. The addition of a P-gp inhibitor leads to an increase in intracellular doxorubicin accumulation in P-gp-overexpressing cells. Applying this approach to identify novel substrates and inhibitors of P-gp, we identified bidwillon A and miltirone as promising candidates. CEM/ADR5000 cells were not resistant, or they were minimally cross-resistant (<2-fold), to both phytochemicals, i.e., sensitive and resistant cells were both efficiently killed by these compounds. In addition to the beneficial growth inhibitory properties of these compounds, they also inhibited P-gp in a similar, or even better manner, as compared to the control drug, verapamil. Speculating that bidwillon A and miltirone would find their way into the clinic to treat multidrug-resistant cancers, they would probably be used as parts of combination therapy protocols, together with standard anticancer drugs, rather than as monotherapies. In this scenario, bidwillon A and miltirone might serve as “two-in-one” drugs. Firstly, they kill MDR cells in a comparable manner to sensitive tumor cells, and secondly, they improve the efficiency of standard anticancer drugs by P-gp inhibition.

As a next step, we were interested in the binding of these compounds, not only to wildtype P-gp, but also to P-gp mutants. While P-gp mutations have been very rarely reported in clinical tumor biopsies during the past three decades [64], assuming that P-gp mutations are clinically of minor interest, this point of view recently changed. By RNA-sequencing of tumor biopsies and additional mining of the internet-based tumor database cBioportal [65], containing RNA-sequencing data, we reported a panel of prior unrecognized mutations in the P-gp-encoding *ABCB1/MDR1* gene [26]. On the other hand, the question whether or not point mutations may affect P-gp’s drug efflux intrigued oncologists and basic scientists alike during all these years. Indeed, altered drug resistance levels were observed in artificial P-gp mutants experimentally generated by in situ mutagenesis [66,67]. Among the novel mutations we identified in clinical tumor samples, there were nonsense and missense mutations [26]. Nonsense mutations lead to truncated, non-functional P-gp mutants that are presumably not capable of correct drug efflux, thereby rendering tumor cells sensitive to chemotherapy. Thus, the diagnosis of nonsense-mutated, truncated P-gp forms may represent a novel strategy for individualized therapy.

The situation with missense mutations is less clear and has been, therefore, addressed in the present investigation. Our molecular docking studies did not show major differences between wildtype and mutated P-gp forms. This is good news, at first sight, as these results indicate that P-gp inhibition by bidwillon A and miltirone may not be compromised by mutations. Nevertheless, the results require a closer look at and comparison with the literature. Our molecular docking analyses demonstrated that the four compounds (i.e., bidwillon A, miltirone, tariquidar, and doxorubicin) neither bettered nor worsened binding to the wild-type and mutated P-glycoproteins. Most of the mutations reported here have been investigated in vitro in baculovirus-transduced HeLa cell models [68]. These mutants did not show inhibited drug-binding, which is consistent with our results, wherein four compounds (i.e., bidwillon A, miltirone, tariquidar, and doxorubicin) neither, for better nor for worse, binded to the mutated and non-mutated P-glycoprotein forms. Instead, the P-gp mutants inhibited ATPase activity of P-gp, leading to an activity stimulation [66]. These authors also generated double and triple mutants, which lost their ability to label P-gp with a transport substrate [69], indicating that the assembling of more than one single mutation may enhance the disturbance of P-gp’s function.

Another feature we recognized not only in the present study, but also previously [26,35], was that compounds bound to the same or closely neighbored binding domains at the inner channel of the transmembrane region, but not to differing amino acid residues. Various reasons can be assumed to explain this observation. The AutoDock program uses simplified force fields, which are useful to predict the binding to rigid protein structures, but changes in the entire ensemble of complex conformational movements cannot be represented [70]. This may also explain that the binding affinities of the compounds in the closed conformations were higher than in the open ones. Closed conformations might stabilize the binding, thereby leading to higher binding affinities, while open conformations may favor the release of compounds into the extracellular space. Another aspect is the poly-specificity of P-gp determined by the amino acid composition around the drug-binding site, as bound ligands are mostly non-specifically coordinated by hydrophobic residues instead of specific hydrogen bonds [71]. Moreover, secondary binding sites may occur if the first binding site is mutated [69]. P-gp is also able to bind multiple ligands at the same time, and each substrate binds to its own residues that may differ from other substrates [72]. Furthermore, as the glycine and proline contents are higher in P-gp compared to other members of the ATP-binding cassette family, P-gp has a higher structural flexibility [73]. High glycine contents provide high degrees of freedom for the backbone dihedral angles [74]. Thus, the high glycine content in P-gp may facilitate an easier transition between different conformations.

In conclusion, by using a combination of in silico and in vitro techniques, we identified bidwillon A and miltirone as candidate compounds from a panel of 375 phytochemicals that may inhibit wildtype and mutated P-glycoproteins. There are two directions that can be discussed to continue from here. One direction is to use extracts of the plants where miltirone and bidwillon A have been isolated from (*Salvia miltiorrhiza* and *Erynthia sigmoidea*, respectively) for the reversal of P-gp mediated multidrug resistance in vivo and in the clinic. This represents a classical phytotherapeutic approach. Another approach is that miltirone and bidwillon A, as prototypes of natural origin, should be used for chemical modification and structure–activity relationship (SAR) studies to improve its P-gp-inhibiting properties and to drive forward the development of clinical candidates. Miltirone derivatives have been synthesized in the past and subjected to SAR studies in different contexts [75,76,77]. These studies may be taken as a proof-of-principle that at least miltirone is in general suitable for derivatization and SAR studies.

## 4. Material and Methods

### 4.1. Virtual Screening and Molecular Docking

A small-scale library, consisting of 375 compounds of alkaloid, flavonoid, and terpene origin, was constructed, and these compounds were previously used by our laboratory. Firstly, the library was screened with PyRx 0.9 software [78] to rank the compounds according to their binding strength with human P-gp. The top 10 compounds with highest and lowest binding energy were selected for the next step: molecular docking with AutoDock 4.2.6 (Molecular Graphics Laboratory, La Jolla, CA, USA) to validate the binding energies. The structure of human P-gp was obtained by homology modelling of the X-ray crystallography-based structure of the mouse P-gp (PDB ID: 4M1M) template [79]. For the modeling of human P-gp mutants, we used one P-gp structure in closed conformation (PDB ID: 6QEX) and another one in open conformation (PDB ID: 4M1M). Heterogeneous atoms (designated as “HETATOMS”) were deleted. Homology modelling was performed by aligning protein sequences in EMBOSS Needle and by using the MODELLER software embedded in UCSF Chimera software (www.cgl.ucsf.edu/chimera/; accessed on 7 January 2023).

Homology models for the mutated ABC transporters were generated by using the wildtype structure as template and exchanging the mutated amino acid sequence. For each mutation, the given amino acid sequence was added, and five new structures were generated using the Swiss-MODEL structure assessment tool. The structure with the lowest z-value was selected as “.pdb” and converted to “.pdbqt” for further molecular docking. Based on the wildtype conformations, open and closed conformations were generated in parallel for all mutated P-gp forms. A detailed description of homology modelling and molecular docking has been previously reported [80].

The grid map for defined molecular docking covered the drug-binding site [71]. Molecular docking was performed using the high-performance supercomputer MOGON (hpc.uni-mainz.de; www.cgl.ucsf.edu/chimera/; accessed on 7 January 2023). Each docking was performed three independent times, consisting of 2,500,000 calculations and 250 runs each. The Lamarckian Genetic Algorithm was chosen for the docking calculations. The corresponding lowest binding energies (LBE) were obtained from the docking log files (dlg) and transferred to an Excel sheet. Dockings with a LBE of −7 kcal/mol were considered to be successful dockings [81]. Mean and standard deviation of binding energies were calculated from the independent dockings. Discovery Studio Visualizer (21.1.0.20298) was used for visualization of docking results. Pearson correlation coefficients and *p*-values were calculated using Excel. Furthermore, the binding site residues of Pgp were compared to the binding site residues of the co-crystallization [71] to reassure that the binding pocket of Pgp was docked.

### 4.2. Torch3D Screening

The StarDrop software (Optibrium Ltd., Cambridge, UK; https://optibrium.com/stardrop/; accessed on 7 January 2023) was used for the structural alignment of compounds with known P-gp inhibitors. This was performed with the Torch3D module. The 3D structures of compounds and the reference molecule were uploaded. The program determined the molecular fields around each compound in their bioactive conformation and compared it to the reference compound. The output score was between “0” and “1” for each match, “1” meaning perfect alignment.

### 4.3. Toxicity Prediction

The testing of potential side effects is an indispensable part in any drug development process with either synthetic compounds or natural products. Therefore, we aimed to predict possible toxic effects with an in silico tool. The toxic potential of all compounds was predicted with Derek (Optibrium Ltd., Cambridge, UK), a knowledge-based toxicity predictor. A hazard for each compound was predicted, meaning the possibility of a chemical causing harm. Reasoning levels within the Derek nexus were: “certain”—the proposition is known to be true, “probable”—there is at least one strong argument for the proposition and none against it, “plausible—the weight of evidence supports the proposition, and “equivocal”—there is an equal weight of evidence for and against the proposition. If no evidence of toxicity has been found, “no report” was returned as output [82].

### 4.4. Cell Culture

All experiments were performed with drug-sensitive CCRF-CEM and multidrug-resistant CEM/ADR5000 leukemia cell lines, which were obtained from Dr. Axel Sauerbrey (Department of Pediatrics, University of Jena, Jena, Germany). The generation of CEM/ADR5000 cells has been described [83]. Cells were maintained in RPMI 1640 medium (Invitrogen, Waltham, MA, USA) and supplemented with 10% fetal bovine serum (FBS) and 1% penicillin/streptomycin. Cells were cultured at 37 °C in a humidified 5% CO_2_ atmosphere. The resistant cells exert the MDR phenotype and overexpress the drug efflux pump P-glycoprotein [84,85]. To maintain P-gp expression, 5 µg/mL doxorubicin were added to culture medium, incubated overnight, and removed by centrifugation to add fresh medium. This procedure was repeated in two-week intervals. For doxorubicin uptake assays, RPMI 1640 phenol red free medium without L-glutamine (Invitrogen) was used.

### 4.5. Resazurin Cell Viability Assay

Cell viability assays were performed, as previously described [86]. Briefly, cells were seeded in 96-well microtiter plates at a concentration of 20,000 cells/well in culture medium (100 µL/well). The compound of interest was adequately diluted, and 100 µL of drug solution were added. The plates were incubated at 37 °C for 72 h. Subsequently, 20 µL of 0.01% resazurin solution was added to all wells and incubated for 4 h at 37 °C. Fluorescence was measured in a TECAN infinite M200PRO photometer (Tecan Trading AG, Männedorf, Switzerland) with a 544 nm excitation/590 nm emission filter set. Experiments were carried out at least three times. The IC_50_ values were calculated using a linear regression model in Excel and are presented as mean values. All in vitro tested compounds were purchased from Sigma-Aldrich, Taufkirchen, Germany and had a purity of >98%. All compounds have been solved in 1% DMSO. This DMSO concentration did not affect the cell viability.

### 4.6. Flow Cytometry

The uptake of doxorubicin with and without the candidate compound was measured by flow cytometry as described [87]. The cytotoxic model substance doxorubicin has distinctive natural fluorescence properties, which can be used to monitor the doxorubicin uptake by cells. CCRF-CEM and CEM/ADR5000 cell suspensions at a density of 10^6^ cell/well were centrifuged at 1200 rpm for 5 min. The supernatant was discarded, and the pellet was resuspended in phenol red-free RPMI 1640 medium. The cells were seeded in 12-well plates at a cell density of 10^6^ cells/well in phenol red-free medium (1 mL/well). The cells were exposed to IC_50_ concentrations of each compound alone and in combination with 20 µM doxorubicin. As a control, one well was only supplemented with 0.5% DMSO, 20 µM doxorubicin, and 20 µM doxorubicin plus 20 µM verapamil, respectively. Verapamil served as a control drug, since it is a known inhibitor of P-gp [88]. After incubation for 6 or 24 h, the cells were centrifuged to discard the old medium. After a washing step with PBS, cells were resuspended in fresh phenol red-free medium. The measurements were performed with a BD Accuri^TM^ flow cytometer using a FL3-A filter (Becton-Dickinson, Heidelberg, Germany). A total of 10,000 living cells were counted per sample. Dead cells were eliminated by gating the living cells with the FSC/SSC scatter. The FL3-A filter was used to detect the fluorescence of doxorubicin.

## Figures and Tables

**Table 1:** Selection of six candidate compounds based on their interaction with P-gp (as determined by AutoDock and PyRx) and their structural alignment with known P-gp inhibitors (as determined by Torch3D). Scores > 0.5 were labeled in bold.

Compound	P-gp Interaction					Torch3D Analysis					
	Defined mode (AutoDock)	Blind Mode (PyRx)	Clomipramine	Verapamil	Lidocain	Trifluoperazine	Valspodar	Tamoxifen	Propanalol	Reserpine	Tariquidar
**Flavonoids:**											
Bidwillon A	−9.0	−9.0	**0.6311**	**0.5551**	**0.6711**	**0.6435**	0.3237	**0.6063**	**0.6763**	**0.5544**	**0.5170**
Neobavaisoflavone	−9.2	−7.9	**0.5918**	**0.5228**	**0.5579**	**0.6147**	0.3632	**0.5825**	**0.5996**	**0.5390**	0.4852
**Alkaloids:**											
Coptisine	−9.0	−9.0	**0.6164**	**0.5666**	**0.5851**	**0.6185**	**0.6148**	**0.5381**	0.2949	0.4526	**0.6024**
**Terpenes:**											
Chamazulene	−8.3	−6.3	**0.5150**	0.2559	**0.6204**	0.2657	**0.6476**	**0.5223**	**0.6289**	0.4540	**0.6225**
Miltirone	−10.0	−7.1	**0.5046**	0.3069	**0.5965**	0.2952	**0.6160**	**0.5874**	**0.6280**	0.4940	**0.6674**
z-Guggulsterone	−9.2	−7.8	0.4904	0.2942	**0.5516**	0.2720	**0.5176**	**0.5645**	**0.5116**	0.4808	**0.5038**

## Data Availability

Data are available upon reasonable request.

## Supplementary Materials

The supporting information can be downloaded at: https://www.mdpi.com/article/10.3390/ijms241210240/s1.
