# Supplementary figures and images for: In Silico and In Vitro Identification of P-Glycoprotein Inhibitors from a Library of 375 Phytochemicals
Source: Int J Mol Sci. 2023 Jun 16;24(12):10240. doi: 10.3390/ijms241210240 (PMC10299530; doi:10.3390/ijms241210240)

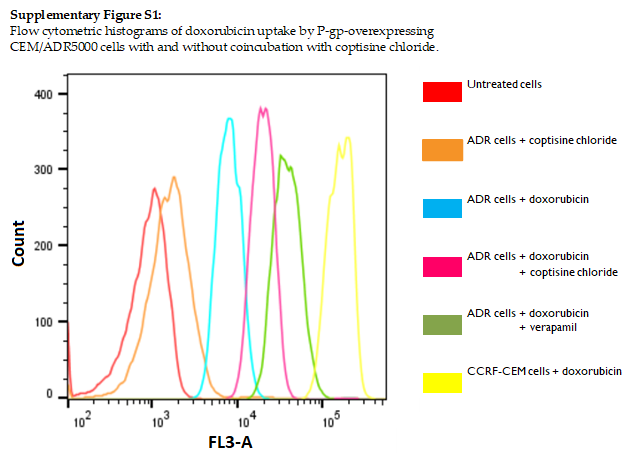

Supplement: Supplementary file 1 [file ijms-24-10240-s001.zip › ijms-2434664-supplementary.png]
